# Does Cataract Surgery Improve the Progression of Age-Related Macular Degeneration? A Meta-Analysis

**DOI:** 10.1155/2020/7863987

**Published:** 2020-09-27

**Authors:** Yuanyuan Liu, Qinhua Cai

**Affiliations:** Department of Ophthalmology, The First Affiliated Hospital of Soochow University, 188 Shizi Street, Suzhou, Jiangsu 215006, China

## Abstract

**Purpose:**

Cataract and age-related macular degeneration (AMD) are the common causes of blindness in the elderly. Although cataract surgery is the most effective treatment for cataract, some clinicians suspect that such interventions may accelerate the progression of AMD. Therefore, we carried out this meta-analysis to focus on demonstrating the effectiveness and safety of cataract surgery in eyes with AMD.

**Methods:**

We performed a systematic literature search in the PubMed, EMBASE, and Cochrane Library databases, and the electronic databases were last searched in January 2019. We planned to include cohort trials of eyes affected by both cataract and AMD in which cataract surgery would be compared to no surgery. Two reviewers independently evaluated the search results against the inclusion and exclusion criteria. 8 trials were included for this meta-analysis.

**Results:**

We used the Stata/12.0 to integrate the data that was extracted from the articles. Eight cohort trials with data from different study populations were included. In random effects model, the relative risk (RR) for the progression of AMD is 1.194 (95% CI 0.897–1.591). As for those grouped according to the follow-up year, the RR for longer than five years was 1.372 (95% CI 1.062–1.772).

**Conclusion:**

We could draw out such a conclusion that there is still a positive correlation between cataract surgery and the progression of AMD, especially for the Asians. However, based on the current results, it is not possible to draw conclusions from existing studies on the impact of cataract surgery on early AMD development.

## 1. Introduction

Cataract and age-related macular degeneration (AMD) are both common causes of impaired visual acuity and blindness in the elderly population worldwide. Cataract progression is the most widespread cause of blindness worldwide [1]. Once vision loss occurs, cataract surgery is the primary treatment that can clearly eliminate lens opacity. Blindness from cataract is very rare in developed countries where it is relatively easy to perform cataract surgery, but AMD remains the second leading cause of visual impairment in the elderly [2]. Cataracts can be treated by removing the opaque lenses to improve vision, and exudative AMD can be treated by intravitreal injection of anti-VEGF, but there is still no effective treatment for the dry form of AMD.

In the past decades, it has become apparent that vision is not sufficient as a criterion for judging whether and when a cataract surgery should be performed on a patient. Concern has been raised that cataract surgery may increase the risk of incident AMD or progression of preexisting AMD. Early histological examinations [3], in which the pathology of the eyes was examined, reported a higher incidence of neovascular AMD in pseudophakic eyes than in lens eyes. As early as the end of the 19th century, a case report [4] reported the exacerbation of senile macular degeneration from a nonexudative to an exudative phase after cataract extraction. Some prospective or retrospective studies [5–7] reported that progression of AMD occurred more often in the surgical eyes compared with the nonsurgical eyes. Some studies [8–11], including systematic reviews [11], have not found a clear correlation between cataract surgery and AMD by comparing AMD patients with cataract surgery and those without AMD. A cross-sectional [12] study has explored this problem, but there are still no exact results.

Cataract and AMD often coexist in patients. The presence of AMD may adversely affect the visual outcome after cataract surgery. However, deferring surgery for visually significant cataract in patients with AMD will also negatively influence the visual function of patients. At the same time, case reports and cohort studies have raised the concern that cataract surgery may increase the risk of progression of AMD. So, how do we advise a patient with visually significant cataract and AMD?

The effect of cataract surgery on progression of AMD was previously evaluated by a review [11] which only included data from published randomized controlled trials. Thus, we found it reasonable to review the literature to include data from cohort trials.

## 2. Methods

### 2.1. Search Strategy

This meta-analysis was conducted following the PRISMA guidelines [13]. We search for keywords such as “macular degeneration, wet macular degeneration, choroidal neovascularization, geographic atrophy or age-related macular degeneration” and “phacoemulsification, cataract surgery, pseudophakia, intraocular lenses, postcataract aphakia or cataract extraction.” We attempted to include cohort studies which were published to date on the relationship between cataract surgery and AMD. All eligible articles were identified by searching the electronic literature PubMed, EMBASE, and Cochrane library databases (latest search update January 2019, covering cataract surgery and AMD). The references lists of reviews and retrieved articles were hand-searched at the same time. All references of the selected articles were reviewed to identify other eligible publications and the author was contacted if necessary. We included only English articles.

### 2.2. Study Selection

1. Type of study: we only included cohort studies comparing the visual performance of AMD patients with and without cataract surgery.
2. Object of study: patients with age-related cataract who had phacoemulsification or with both cataract and AMD were included; patients with a history of surgery that may affect postoperative vision outcomes may be excluded.
3. Outcome measures: describe whether cataract surgery will increase the risk (relative risk (RR)) of AMD progression.

### 2.3. Data Extraction

Two independent reviewers used the same standardized form to extract data. The reviewers applied the Newcastle-Ottawa scale to assess risk of bias in nonrandomized studies [14]. Information obtained from each full-text study included first author, publication year, study design, number of control and case groups, location, AMD classification, follow-up time, RR/odds ratio (OR)/hazard ratio (HR) and 95% CI, and patients' age. When several estimates were available, we used the one that was adjusted for the most variables. If there were any discrepancies, they would be resolved by discussion.

### 2.4. Assessment of Methodology Quality and Statistical Analysis

Each study estimated the relationship between cataract surgery and the progression of AMD. Considering the low prevalence of AMD, we can generally ignore the distinctions among the various measures of relative risk (e.g., odds ratios, hazard ratio. and risk ratios) [15]. We derived summary estimates of the RR for each study using both fixed effects models and random effects models [16]. Only the results from the latter models which take into account both within-study and between-study variability were, however, presented in order to take into account the heterogeneity of risk estimates (thus being more conservative). We assessed the heterogeneity between studies using the *χ*^2^ test (defining a significant heterogeneity as a *p* value < 0.10) [15]. Sensitive analysis was also performed to evaluate the influence of individual studies on the final effect. Potential publication bias would be observed by the funnel plot quantified by Egger's and Begg's tests [17, 18]. An asymmetric plot indicates a possible publication bias; otherwise, the plot should be shaped like a funnel. Subgroup analyses were executed based on location, follow-up time, and AMD classification which was conducted regarding the association of AMD and cataract surgery, respectively. To assess the impact of individual studies on aggregated results, sensitivity analysis was performed by excluding individual studies in turn to test the stability of our study. All the statistical analyses were performed using Stata 12.0 software. *p* < 0.05 was considered statistically significant, except where otherwise specified.

## 3. Results

### 3.1. Literature Search

**Figure 1:**
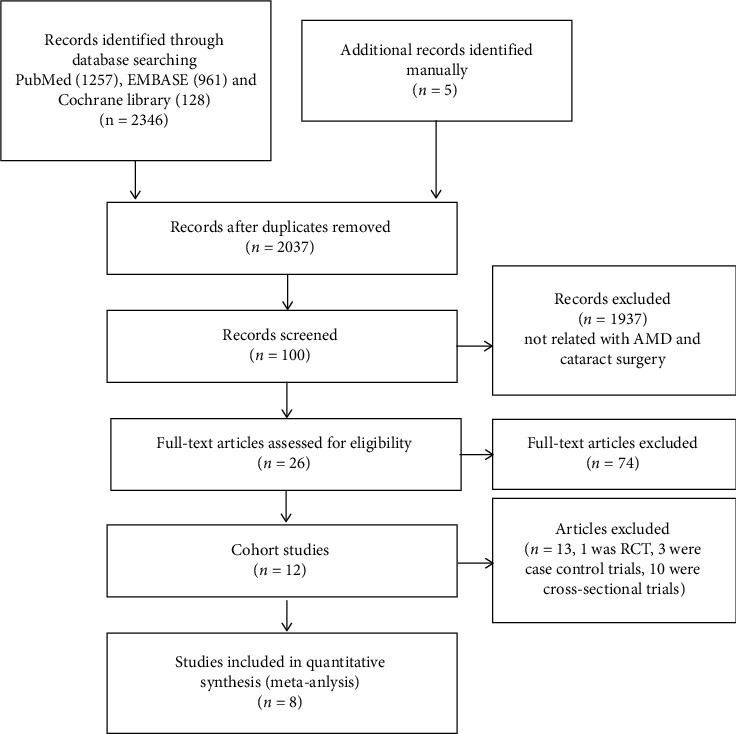
The trials selection process.

A total of 2,346 documents from multiple databases and 5 documents identified manually were retrieved, and 2,037 documents remained after the duplicates were deleted. After deleting the documents which were unrelated to cataract surgery and AMD progression based on the titles and abstracts, there were remaining 100 articles. All trials describing whether cataract surgery will increase the risk of AMD progression can be included. After reading the full text, 26 articles were selected, of which 1 was RCT, were case control trials, 10 were cross-sectional trials, and the rest were cohort trials. We only chose the cohort trials in quantitative synthesis (meta-analysis). To reduce duplicate synthesis in the same control group, for the same study population, we select the most recently published literature. The trials selection process is shown in Figure 1.

### 3.2. Characteristics of Eligible Studies

The characteristics of the trials included in the current meta-analysis are shown in Table 1. Two studies [19, 20] were conducted in Europe; two studies [21, 22] were conducted in Asia; others [6, 23–29] were from America and Oceania. All patients in the study were older than 42 years and were followed from three months to twenty years, as shown in Table 1.

## 4. Quality Assessment

Table 2 evaluates the cohort studies using the coding manual for cohort study. The quality of the included articles was evaluated from three aspects, such as collection, comparability, and outcome. The total score of the coding manual is 10 points. If the score is greater than or equal to 8, the article is a high-quality article. As is shown in Table 2, all of the articles are high-quality literature.

### 4.1. Efficacy Analysis

**Figure 2:**
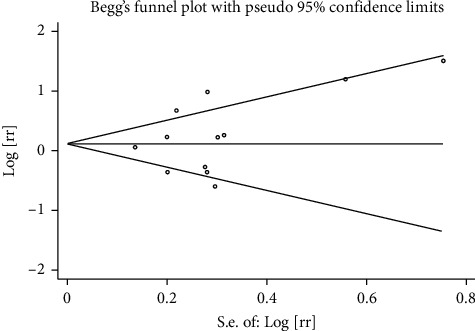
The funnel plot in Egger's test and Begg's test.

**Figure 3:**
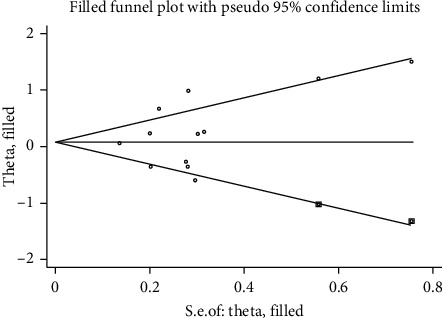
The filled funnel plot.

**Figure 4:**
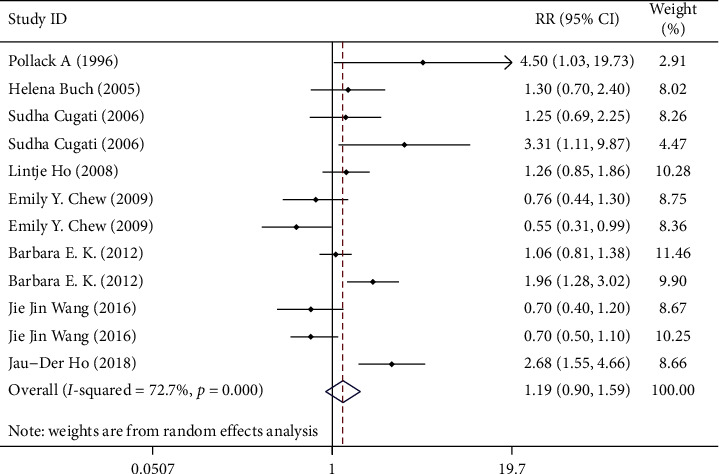
The RR for cataract surgery and AMD in the random effects model.

**Figure 5:**
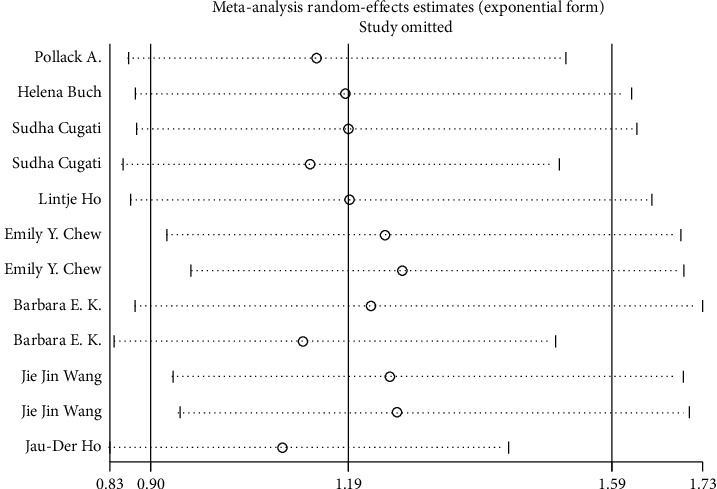
Sensitivity analysis.

We use the Stata/12.0 to integrate the data that was extracted from the articles. Eight studies with data from different study populations were included. We use RR to indicate the relationship between cataract surgery and AMD progression. Some studies have reported that cataract surgery can aggravate the progression of AMD, although the intensity of associations varies from study to study, and some small studies report a particularly strong inverse association. It can be seen from the visual inspection of the funnel plot that there is no publication bias (Egger's test Pr > |*t*| = 0.323 (95%CI-1.78–4.89); Begg's test Pr > |*z*| = 0.373) (the results are shown in Figures 2 and 3). The summary estimate RR was 1.21 (95% CI 0.978–1.290). Though this result cannot show that cataract surgery is one of the risk factors of AMD, the data has high heterogeneity (*I* squared = 72.7%) and the difference was not statistically significant (*z* = 1.65, *p*=0.100), so we should use random effects model to calculate data. In random effects model, the RR for the development of AMD is 1.194 (95% CI 0.897–1.591). The detailed data are shown in Figure 4. The result explains that the data is heterogeneous (*I* squared = 72.7%), and the difference was not statistically significant (test of RR = 1: *z* = 1.21, *p*=0.225). When performing sensitivity analysis, we found that it is low sensitivity and the result is more stable and credible (Figure 5). There are a lot of risk factors for AMD, such as age, gender, and smoking history, and the trials we included were all adjusted for age and gender. The heterogeneity between the studies is large, which may result from difference in location, follow-up time, and AMD classification. As far as the current data is concerned, the results require further group integration of RR.

**Figure 6:**
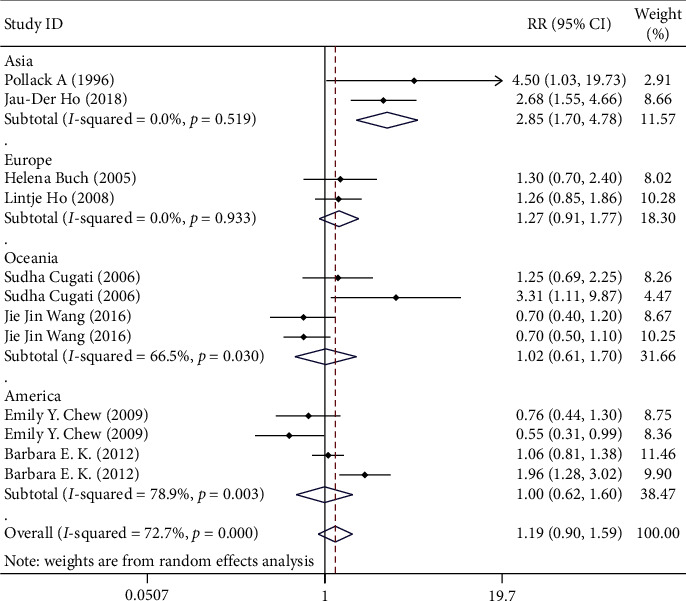
Subgroup analysis on different regions.

It can be divided into four groups according to the locations, which are Asia, Europe, Oceania, and America. For those grouped according to the location, the RR for Asia was 2.855 (95% CI 1.704–4.781), Europe was 1.271 (95% CI 0.914–1.769), Oceania was 1.017 (95% CI 0.607–1.703), and America was 0.997 (95% CI 0.621–1.601). As shown in Figure 6, for studies where the location is in Asia, the results showed a significant correlation between cataract surgery and the progression of AMD. However, studies in other locations cannot show that cataract surgery can accelerate the development of AMD.

**Figure 7:**
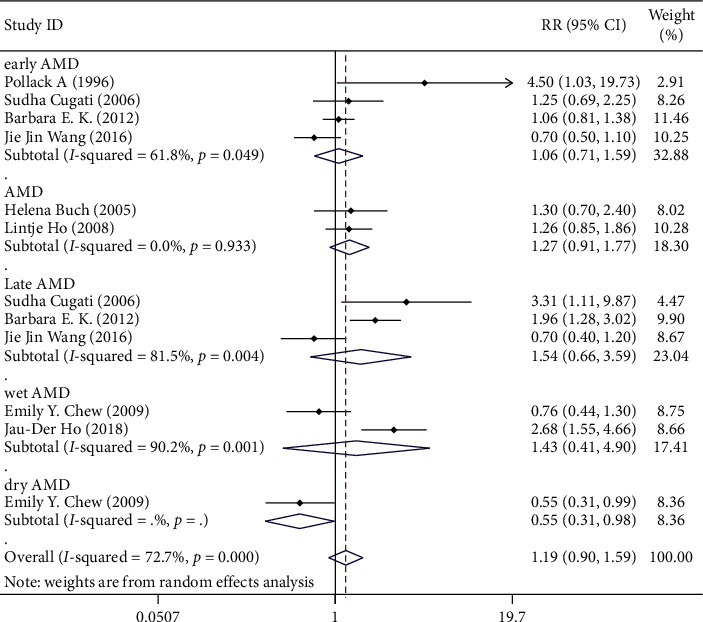
Subgroup analysis on different AMD classification.

According to the records of different data in different documents, they can be divided into three groups according to the classification of AMD, which are, respectively, early AMD, late AMD, and AMD. For those grouped according to the classification of AMD, the RR for early AMD was 1.059 (95% CI 0.706–1.591), late AMD (including wet AMD and dry AMD) was 1.254 (95% CI 0.705–2.232), and AMD was 1.271 (95% CI 0.914–1.769). As shown in Figure 7, though the RR values are across the vertical line, there is not a positive correlation between the cataract surgery and progression of AMD. The differences between the groups were not statistically significant, so we can conclude that the classification of AMD is not the cause of heterogeneity.

**Figure 8:**
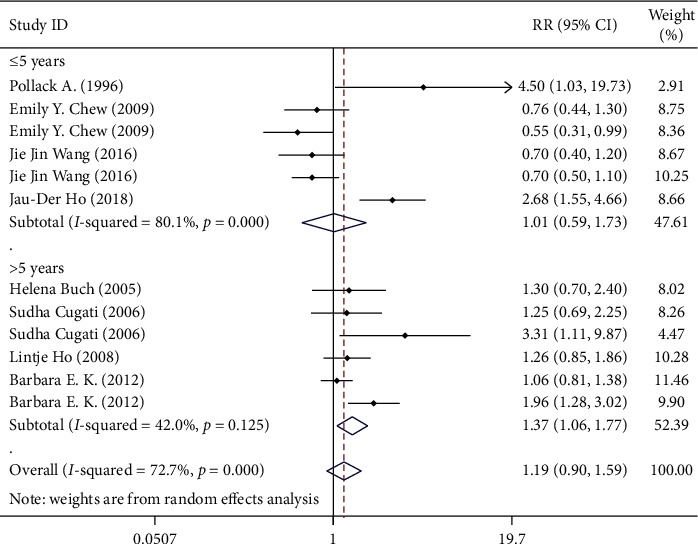
Subgroup analysis on follow-up time.

According to the follow-up time classification, they can also be divided into 2 groups. For those grouped according to the follow-up year (Figure 8), the RR for less than 5 years or 5 years was 1.011 (95% CI 0.592–1.728) and RR for longer than 5 years was 1.372 (95% CI 1.062–1.772). As the follow-up time increases, the correlation between the cataract surgery and progression of AMD becomes more and more obvious.

## 5. Discussion

Many large epidemiologic studies do not provide a clear indication of whether cataract surgery is associated with an increased risk of AMD progression. The aim of our meta-analysis was to determine the effect of cataract surgery on the progression of AMD and provide evidence-based recommendation on the care of patients with coexisting AMD and cataract. Only a small number of publications could be included in the article. It needs to be mentioned that randomizing patients to not undergo cataract surgery when their vision is poor enough to affect their daily life would neither be ethical nor practicable.

From the results in this article, it can be found that Asians are more likely to develop AMD after surgery. This is similar to the study in South Korea [22], which shows that the incidence of neovascular AMD after cataract surgery within five years is 2-3 times that of nonoperative patients (HR: 2.68; 95% CI 1.55–4.66; *p* < 0.01). Asians and Caucasians differ in AMD genetic structure [30]. There are many studies on Europe and America, and relatively few studies on Asia [10, 22, 31] and Africa [12]. Lazreg et al. [12] found in a cross-sectional study of North Africans living in Algeria and Italy that patients with cataract surgery are more likely to have AMD than those who have not had surgery (OR: 2.69; 95% CI 1.96–3.70; *p* < 0.0001). Darker irises are also related to AMD, and it may affect the relationship between the surgery and AMD [31]. There should be more population-based prospective studies on other regions to explore such issues.

For this meta-analysis, AMD severity was classified as early AMD and late AMD (including wet AMD and dry AMD). Thus, the classification could be applied to all included studies, enabling a reasonable comparison of the individual studies. From the results of subgroups classified by AMD type, we could not conclude that cataract surgery can aggravate all types of AMD (RR: 1.271; 95% CI 0.914–1.769), and there is a high degree of heterogeneity in early and late AMD, and the results are not statistically significant. The included studies showed noticeable differences in the duration of the follow-up and did not present reasons for the chosen duration. When the follow-up time is longer than 5 years, there is an exact relationship between cataract surgery and AMD, and the RR is 1.372 (95% CI 1.062–1.772). Many studies have found that different clinical subtypes of AMD have different risks for developing neovascular AMD. Combining the results of Beaver Dam and Blue Mountain, it was found that cataract surgery was related to five-year incidence of neovascular AMD [23]. The 10-year follow-up result of Beaver Dam also showed that cataract surgery can increase the risk of late AMD [28]. Klein et al. also found that the OR for late AMD was higher for cataract surgery performed five years or more than five years prior compared with less than five years prior [29]. The epidemiological studies offered incidences for AMD after up to 20 years of follow-up, whereas the clinical trials had a follow-up time of no more than one year. Overall, the included studies showed considerable differences concerning study population and study period; hence, their comparability was limited. However, only cohort studies were included; thus, we believe our results are valid, although we could not identify individual risk factors. As the follow-up time is longer than five years, the correlation between cataract surgery and the progression of AMD becomes stronger. Many studies on long-term follow-up of patients with AMD have found that they are at a higher risk of developing the progression of AMD with cataract surgery than without [6, 28]. As it is shown, those who reported surgery five or more years earlier had 2.1 times the odds of late AMD (95% CI 1.0–4.6) and who underwent surgery less than five years earlier had modestly elevated odds of late AMD, although it was not statistically significant (OR = 1.4, 95% CI 0.7–2.6) in a cross-sectional study combining three population-based studies [32]. One population-based cohort of older Australians reported that there is a higher long-term (10-year) risk of developing late AMD in the eyes which underwent surgery than in phakic eyes at baseline [6]. The 10-year follow-up Blue Mountain eye study shows that there is a higher risk of developing both neovascular AMD (RR: 4.3; 95% CI 1.7–10.9) and geographic atrophy (RR: 3.2; 95% CI 1.3–7.6) in nonphakic than phakic eyes [28]. At the same time, it is important that patients with drusen or pigmentary changes have a risk of developing late AMD with or without cataract surgery. Ferris et al. [33] found that the risk of developing late AMD is as high as 50% in five years for patients with intermediate AMD (bilateral large drusen) or with unilateral advanced AMD. In summary, whether or not cataract surgery is performed, it is necessary for patients with AMD to regularly undergo fundus examination.

Although we did not find a clear link between cataract surgery and AMD, some studies have found that cataract and AMD may have the same risk factors epidemiologically [32, 34]. Therefore, it has also been proposed that cataract surgery may only be a surrogate indicator of cataract severity [28]. Both cataract and AMD are strongly age-related, but there is no consistent evidence to suggest that they are directly related or that they share the same etiology [34]. Some reports [32, 35] reported that there is a significant association between cataracts and AMD. One case-control study showed that cloudy lenses are significantly associated with all types of AMD [36] and one trial [34] reported that nuclear sclerosis is associated with early AMD but not with late AMD (geographic atrophy), although other trials [5] reported the relative risk of AMD to be lower than 1.00 in the presence of nuclear sclerosis. As the possible interaction of AMD, cataract, and cataract surgery did not seem to be all coherent, residual confounding by the cataract status might be possible, even though most included observational studies did control for different familiar confounders. However, all of these are not enough to explain the correlation between cataract surgery and the progression of AMD presented in this study.

Cataract surgery accelerates the progress of AMD, and the following mechanisms may exist. First, the elderly's lens effectively absorbs short wavelengths thereby providing protection against short-wavelength irradiation. The natural lens is removed and replaced with an artificial intraocular lens (IOL) that provides less protection against short wavelengths [37] after cataract surgery. There is evidence that acute exposure to short-wave (UVB and UVA) radiation can damage the retina [30]. Experimental evidence [38] suggests that blue-light-blocking lenses could theoretically benefit patients with AMD. Patients who have not implanted an anti-blue-light/ultraviolet lens have higher risk of developing AMD after surgery, because of the damage to the retina due to the changes in intraocular (free radicals) caused by light damage [35]. At the same time, the increase of light exposure of retina after removing the lens including light toxicity during the operation [39] may explain a possible positive relationship between cataract surgery and AMD. These indirectly illustrate the rationality of blue light toxicity. Therefore, chronic exposure to the blue wavelengths in sunlight may damage the retina and increase the risk of AMD progression.

Second, cataract surgery may directly impact progression of early AMD, such as photic retinal injuries caused by the operating microscope [40], mechanical damage, or intraocular pressure change causing trauma to the retina and/or choroid [3]. Photic retinal injuries caused by the operating microscope can increase retinal pigmentation and RPE depigmentation which is the sign of AMD [41]. An eye with early AMD may be especially vulnerable to trauma because Bruch's membrane is altered [42], so that mechanical and intraocular pressure change can break Bruch's membrane, making it easier for retinal neovascularization.

The third possible factor is cataract surgery-related, intraocular inflammation is common after cataract surgery. As early as 1994, Van der Van der Schaft et al. [3] proposed the hypothesis that cataract surgery may in some ways make the eye susceptible to AMD through inflammatory mechanisms. Acute or chronic postoperative inflammation [43], complement pathway, macrophage induction [21], and proinflammatory chemokines may act as an additional angiogenic stimulus [44]. When the blood–aqueous barrier is compromised, inflammation and oedema occur which can increase the vascular permeability [45]. However, this mechanism can only explain the short-term effects of cataract surgery on AMD.

Fourth, theoretical link between AMD progression and cataract surgery is related to the immune system and inflammatory response induced by cataract surgery. Increasing evidence points towards imbalance in inflammatory regulation as a hallmark in the pathogenesis of AMD [46] as well as in the progression to neovascular AMD [47]. Manipulation of the immune system could form the basis of a potential future therapy for the dry form of AMD [48]. At least in theory, cataract surgery could upset the immunological balance and thereby increase the risk of progression of AMD although no evidence supports this theory yet.

Last, there may be a genetic factor that is also a mechanism for the development of AMD after cataract surgery [7]. The analysis of this paper based on regional subgroups shows that Asians are more likely to have AMD progression after cataract surgery, and this can be explained by the differences between Asian and White patients in the genetic architecture of AMD [30]. Wang et al. [49] found that homozygous CFH Y402H carriers had higher risks for all types of AMD. Kaiserman et al. [7] believed that physiologic or genetic factors that caused the lens to become opaque may cause macular degeneration. There should be more studies to confirm our findings and explore the mechanisms so that more prevention and treatment measures can be put forward for AMD, which also has more guiding significance for cataract surgery in AMD patients.

This meta-analysis also has some limitations. First, there were no trials at the highest level of evidence, which are randomized controlled trials (RCT) or systematic reviews of RCT, because there are a series of obstacles [42], such as large sample size, and it is difficult to deny patients cataract surgery in eyes randomly allocated to the control group, to carry on a RCT. Therefore, prospective or retrospective studies may provide the strongest evidence. Additional epidemiological and clinical studies that examined the association of cataract surgery and AMD were identified in the literature search, but all of them were cross-sectional studies and explored exposition and outcome simultaneously. Second, the doctor's decision on which patient and which eye to perform the operation is unethical, and the doctor's decision may be biased towards the patient with poor vision, so it is impossible to determine the comparability of the surgery group with the costly surgery group. An observational population-based retrospective case-control study investigated the association between the cataract surgery and AMD by comparing the rate for undergoing PDT at different time periods after cataract surgery [7]. Third, in some studies [50], some patients whose fundus was unclear due to cataracts were excluded, which artificially reduced the prevalence of AMD. So, it may influence the statistical analysis of the associations of AMD with other parameters, because AMD and cataracts have the same risk factors, such as age. The inclusion and exclusion criteria adopted by studies in different centers have resulted in great heterogeneity. Last but not least, physicians may pay more attention to patients with poor vision when deciding whether to have cataract surgery, so we cannot confirm the comparability between the surgical and control groups.

There is no firm conclusion as to whether cataract surgery can promote the progress of AMD. On the one hand, surgeons are concerned about serious visual impairment caused by neovascular AMD after removing the opaque lens. On the other hand, cataract patients with early AMD cannot be excluded from cataract surgery, as there have been quite a few studies [51, 52] that show that patients with AMD have improved visual effects and quality of life after cataract surgery.

## 6. Conclusion

We could draw out such a conclusion that there is still a positive correlation between cataract surgery and the progression of AMD and that cataract surgery increases the progression of early AMD to late AMD as follow-up years increase. The previously mentioned results show that a temporal sequence of cataract surgery and subsequent AMD development or progression would be necessary to conclude a causal relationship. However, according to the current results, it is not possible for us to draw out conclusions from existing studies on the impact of cataract surgery on the development of AMD. Overall, the included studies showed considerable differences concerning study population and study period; hence, their comparability was limited. Therefore, additional clinical trials (with sufficient statistical power) are needed to demonstrate this hypothesis by adequate control of confounding variables such as age and cataract severity. Research hypotheses and possible influencing factors, such as lens type and type of surgery, need to be clearly stated for a comprehensive assessment.

## Figures and Tables

**Table 1:** Characteristics of articles included in this meta-analysis.

Author	Publish year	Study design	No. of cases/control	Location	Adjustment/match	AMD classification	Follow-up	OR/RR/HR	CI	*p*	Age
Pollack	1996	Cohort	47/47	Asia	—	Early AMD	1 year	RR = 4.500	1.027–19.726	0.054	80.4 (67–94)

Lintje Ho	2008	Cohort	6032	Europe	Age, sex, follow-up time, and the correlation between eyes	AMD	5.7 (2.8–9.7)	OR = 1.26	0.85–1.86	—	≥55
						Early AMD		OR = 1.31	0.88–1.95		
						Dry AMD		OR = 3.44	1.68–7.08		
						Wet AMD		OR = 0.93	0.35–2.49		

Jie Jin Wang	2003	Cohort	6019	Oceania and North America (BDES and BMES)	Baseline age, gender, smoking status (current, past, and never), and preexisting early-stage ARM lesions at baseline	Late AMD	5 years	OR = 5.7	2.4–13.6	—	—
						Dry AMD		OR = 4.5	1.4–14.7		
						Wet AMD		OR = 4.9	1.9–12.4		
				North America (BDES)		Late AMD		OR = 7.3	2.6–20.4		
						Dry AMD		OR = 4.7	1.4–16.1		
						Wet AMD		OR = 6.8	1.9–24.3		
				Oceania (BMES)		Late AMD		OR = 4.2	1.0–17.5		
						Dry AMD		OR = 7.6	1.1–55.0		
						Wet AMD		OR = 3.1	0.7–13.2		

Sudha Cugati	2006	Cohort	1952	Oceania (BMES)	Age, gender, smoking	Early AMD	10 years	OR = 1.25	0.69–2.25	—	≥49
					Age, gender, smoking, and presence of early ARM lesions	Late AMD		OR = 3.31	1.11–9.87		
						Dry AMD		OR = 2.34	0.51–10.8		
						Wet AMD		OR = 3.42	1.07–10.91		

Jau-Der Ho	2018	Cohort	3465/10395	Asia	Patient's geographical location, urbanization level, monthly income, diabetes, hypertension, and cardiovascular and hyperlipidaemia	Wet AMD	5 years	HR = 2.68	1.55–4.66	<0.01	70.2 ± 9.6

Helena Buch	2005	Cohort	359	Europe	Age, sex	AMD	14 years	OR = 1.3	0.7–2.4	—	82.4 ± 4.63 (75–95)
						Late AMD		OR = 1.6	0.8–3.2		

Emily Y. Chew	2009	Cohort	6037	America (AREDS)	Age, gender, race, smoking, AREDS treatment group, and eye-specific AMD severity status	Wet AMD	5 years	OR = 0.76	0.44–1.30	0.31	55–80
						Dry MD		OR = 0.55	0.31–0.99	0.047	

Jie Jin Wang	2012	Cohort	1178	Oceania (CSAMD)	Baseline early AMD in models for late AMD outcome (paired comparison)	Late AMD	3 years	OR = 0.74	0.23–2.36		≥65
						Early AMD		OR = 1.07	0.74–1.65		
Jie Jin Wang	2016	Cohort	2029		Baseline early AMD in models for late AMD outcome (paired comparison)	Late AMD	5 years	OR = 0.7	0.4–1.2		
						Early AMD		OR = 0.7	0.5–1.1		

Ronald Klein	2002	Cohort	2764	North America (BDES)	Age, sex, vitamin use, smoking, drinking, and systolic blood pressure	AMD	10 years	RR = 1.97	1.29–3.02	<0.01	43–86
						Early AMD		RR = 1.36	0.82–2.23	0.23	
						Late AMD		RR = 3.81	1.89–7.69	<0.01	
						Wet AMD		RR = 4.31	1.71–10.9	<0.01	
						Dry AMD		RR = 3.18	1.33–7.60	<0.01	

Klein, B. E.	2012	Cohort	3275	North America (BDES)	Age, sex, education, smoking, drinking, cardiovascular disease, diabetes, and diastolic blood pressure	Early AMD	20 years	OR = 1.06	0.81–1.38	0.7	43–86
			3585			Late AMD		OR = 1.96	1.28–3.02	0.002	

Ronald Klein	1998	Cohort	3684	North America (BDES)	Age, smoking, beer consumption, drinking, pulse pressure, hypertension, and vitamin use	Early AMD	5 years	OR = 1.73	0.93–3.21	0.08	43–86
						Late AMD		OR = 2.80	1.03–7.63	0.04	≥65
						Wet AMD		OR = 1.67	0.39–7.18	0.49	≥65
						Dry AMD		OR = 3.49	0.80–15.16	0.1	≥65
						AMD		OR = 2.57	1.61–4.11	<0.001	43–86

**Table 2:** Evaluation of the quality of cohort studies included in the meta-analysis.

Author	Publish year	Study design	Selection				Comparability	Outcome			Score
			Representativeness of the exposed cohort	Selection of the nonexposed cohort	Ascertainment of exposure	Demonstration that outcome of interest was not present at start of study	Comparability of cohorts on the basis of the design or analysis	Assessment of outcome	Was follow-up long enough for outcomes to occur?	Adequacy of follow-up of cohorts	
Pollack	1996	Cohort	1	1	1	1	0	1	1	1	8
Lintje Ho	2008	Cohort	1	1	1	1	2	1	1	1	10
Jie Jin Wang	2003	Cohort	1	1	1	1	2	1	1	1	10
Sudha Cugati	2006	Cohort	1	1	1	1	2	1	1	1	10
Jau-Der Ho	2018	Cohort	1	1	1	1	2	1	1	1	10
Helena Buch	2005	Cohort	1	1	1	1	2	1	1	1	10
Emily Y. Chew	2009	Cohort	1	1	1	1	2	1	1	1	10
Jie Jin Wang	2012	Cohort	1	1	1	1	2	1	1	1	10
Jie Jin Wang	2016	Cohort	1	1	1	1	2	1	1	1	10
Ronald Klein	2002	Cohort	1	1	1	1	2	1	1	1	10
Klein	2012	Cohort	1	1	1	1	2	1	1	1	10
Ronald Klein	1998	Cohort	1	1	1	1	2	1	1	1	10

## Data Availability

No data were used to support this study.
